# Lock-picks: fungal infection facilitates the intrusion of strangers into ant colonies

**DOI:** 10.1038/srep46323

**Published:** 2017-04-12

**Authors:** Enikő Csata, Natalia Timuş, Magdalena Witek, Luca Pietro Casacci, Christophe Lucas, Anne-Geneviève Bagnères, Anna Sztencel-Jabłonka, Francesca Barbero, Simona Bonelli, László Rákosy, Bálint Markó

**Affiliations:** 1 Hungarian Department of Biology and Ecology, Babeş-Bolyai University, Clinicilor 5-7, 400006 Cluj-Napoca, Romania; 2 Department of Taxonomy and Ecology, Babeş-Bolyai University, Clinicilor 5-7, 400006 Cluj-Napoca, Romania; 3 Museum and Institute of Zoology, Polish Academy of Sciences, Wilcza 64, 00-679, Warsaw, Poland; 4 Department of Life Sciences and Systems Biology, University of Turin, via Accademia Albertina 13, 10023 Torino, Italy; 5 Institut de Recherche sur la Biologie de l’Insecte, UMR CNRS 7261, Université François Rabelais, 37200 Tours, France

## Abstract

Studies investigating host-parasite systems rarely deal with multispecies interactions, and mostly explore impacts on hosts as individuals. Much less is known about the effects at colony level, when parasitism involves host organisms that form societies. We surveyed the effect of an ectoparasitic fungus, *Rickia wasmannii*, on kin-discrimination abilities of its host ant, *Myrmica scabrinodis*, identifying potential consequences at social level and subsequent changes in colony infiltration success of other organisms. Analyses of cuticular hydrocarbons (CHCs), known to be involved in insects’ discrimination processes, revealed variations in chemical profiles correlated with the infection status of the ants, that could not be explained by genetic variation tested by microsatellites. In behavioural assays, fungus-infected workers were less aggressive towards both non-nestmates and unrelated queens, enhancing the probability of polygyny. Likewise, parasitic larvae of *Maculinea* butterflies had a higher chance of adoption by infected colonies. Our study indicates that pathogens can modify host recognition abilities, making the society more prone to accept both conspecific and allospecific organisms.

In natural ecosystems, relations among different organisms are characterized by a high degree of complexity which makes studies on multispecies systems difficult. For this reason, many investigations focus exclusively on pairwise relationships^1^,^2^, although the outcomes of these interactions vary and depend on the context^3^. For instance, in the classical case of the brood parasitic giant cowbirds (*Scaphidura oryzivora*), where the presence of other parasites, as botflies, may change the cowbird’s effect on the host from damaging to beneficial^4^. In the amphipod *Gammarus insensibilis* the parasite manipulation is less efficient when the host is infested with both nematodes and trematodes, compared to being exclusively parasitized by trematodes^5^. Increase in immune function caused by the presence of the mildly antagonistic ectosymbiont *Laboulbenia formicarum* might boost the survival chances of the host ant *Lasius neglectus* exposed to the lethal entomopathogen fungus *Metarhizium brunneum*^6^.

Interaction between different organisms could have more complex consequences when social animals, as e.g. ants, are targeted. Changes induced by parasites at the individual level may also be reflected in the social system through the interaction of host individuals. Generally, the structure of social insect colonies, in which many similar individuals live together, makes them attractive for social parasites and various pathogens^7^,^8^. This is especially true in the case of ants due to the stability of their colonies and their mostly ground-based nesting habits, which make their colonies accessible to a plethora of organisms. Among these, fungi are the most important antagonists ants must constantly deal with, and they can occur inside the colony either as ectoparasites on the host cuticle, endoparasites, or endosymbionts^8^,^9^. Some fungi are even able to manipulate host ant behaviour to increase their own fitness^10^. The relationships between the hosts and these fungi often have spectacular outcomes, but as these parasitic species have quite a low prevalence^11^, their interference with other parasites is hard to study. Certain fungi, like *Rickia wasmannii*^9^ an ectoparasitic fungus of *Myrmica* ants, however, are common in some populations with up to 50% of colonies infected, and with high within-colony prevalence^12^. Therefore, they can serve as easily accessible model organisms to test the effects of parasitic infections both on individuals and societies.

Since one of the main communication channels in ant societies is based on surface chemical compounds, including identification of non-kin conspecifics and nestmates^13^, the composition of these chemical substances and the ways in which these substances are perceived may be affected by such epicuticular fungus. Nestmate recognition is a dynamic process, largely based on specific cocktails of cuticular hydrocarbons (CHC) covering the surface of all individuals within a society, the primary biological function of which is to prevent desiccation^7^,^14^. The template model suggests that each individual uses its own cocktail of CHCs to match against others^15^. Social interactions, such as allogrooming, ensure an exchange of these mixtures between nestmates and give rise to a collective CHC *gestalt* odour^1^,^15^. The *gestalt* may be relatively homogenous in small monogynous societies, but it may be blurred, to a certain extent, in large polygynous colonies due to higher genetic diversity^15^ and the higher number of interactions needed to assemble the full colony^16^. Consequently, a margin of error is tolerated when a worker assesses the CHC of another ant, resulting in a context-dependent and dynamic threshold of similarity at which individuals distinguish nestmates from strangers^1^,^17^. Therefore, it is plausible to assume that epicuticular fungal infection may affect accuracy of recognition systems in ant colonies, especially because its prevalence within infected ant colonies can reach extreme values. In certain colonies almost 100% of workers are parasitized and fungal thalli may cover the entire body of its host^12^. Recently, it was demonstrated that the fungus *Rickia wasmannii* not only reduces the lifespan of infected ant workers^18^,^19^, but also causes subtle changes in the host’s behaviour by increasing worker allogrooming frequency, which may enhance parasite transmission^19^. As *Myrmica* ants interact with several other invertebrates and host diverse social parasites^20^, fungus-induced behavioural changes as well as altered nestmate discrimination abilities could shape associations of *Myrmica* ants with other organisms. One example could be the adoption of new queens, since many *Myrmica* ants display facultative polygyny, as a consequence of which foreign queens might be adopted^21^.

Finally, changes in ant chemical cues become essential in the case of the socially parasitic larvae of *Maculinea* butterflies, which infiltrate the ant colony by breaking the nestmate recognition code^22^,^23^,^24^. Various *Maculinea* species use different feeding strategies inside the host colony, preying directly on the ant brood (namely predatory) or being fed by throphallaxis (called cuckoo), and they also exhibit different levels of chemical adaptations to infiltrate and integrate into the ant colony^20^,^23^,^25^. During the adoption, cuckoo larvae, as *M. alcon*, are already chemically similar to their host ants^22^,^23^,^26^, while predatory larvae, such as *M. teleius*, possess a less sophisticated chemical mimicry that they have to compensate for with complex behaviour^27^.

The above multipartite system, involving distantly related organisms ranging from fungus to ants, is a perfect model for the study of how the presence of one partner could alter the infiltration chances of the others, which in turn potentially has consequences for the fitness of all the interacting entities, and for the society as a whole.

The main aim of our work was to assess the effect of *Rickia wasmannii* infection on *Myrmica scabrinodis* host ant behaviour and interaction with other organisms. Specifically, we investigated the differences between infected and uninfected ants (i) in CHC profiles, while exploring the (ii) genetic and social structure of colonies, and the differences (iii) in kin-discrimination ability towards non-nestmates and unrelated queens, and (iv) in adoption rate of two socially parasitic *Maculinea* species, *M. alcon* and *M. teleius*, having diverse feeding strategies, cuckoo and predatory, respectively.

## Results

### The effects of fungal infection on the CHC profile of ants

**Figure 1:**
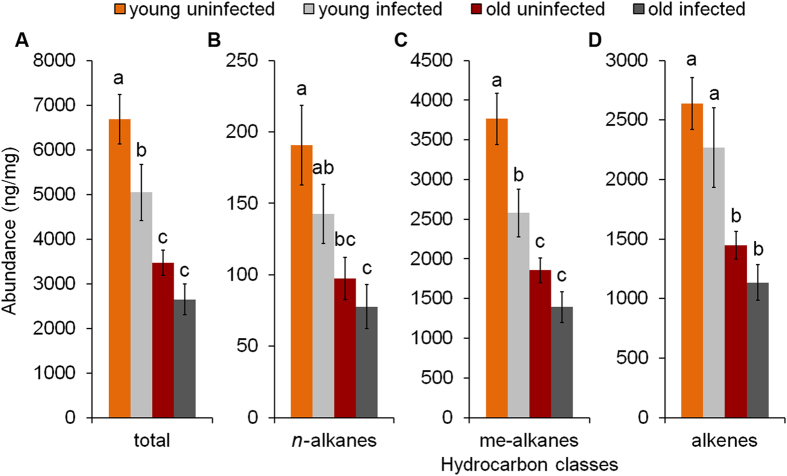
Abundance (±SE) of the CHC profile – (**A**) total, (**B**) alkanes, (**C**) methyl-branched alkanes and (**D**) alkenes – extracted from the body surface of young and old *M. scabrinodis* workers from uninfected and infected colonies. Bars with different letters are statistically different according to LMM pairwise comparisons.

**Figure 2:**
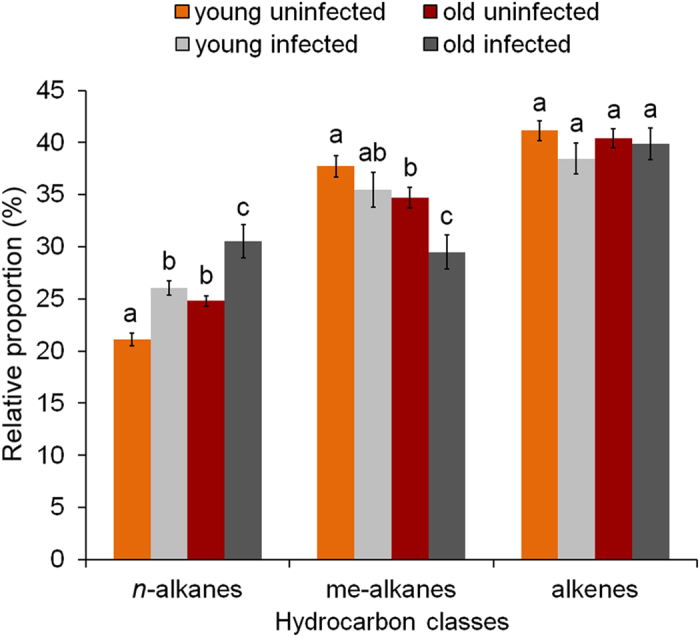
Relative proportions (±SE) of different CHC classes (alkanes, branched alkanes and alkenes) extracted from the body surface of young and old *M. scabrinodis* workers from uninfected and infected colonies. Bars with different letters are statistically different according to LMM pairwise comparisons.

*M. scabrinodis* possesses complex CHC blends that include 37 identified hydrocarbons; these are homologous series of linear alkanes, methyl-branched alkanes and linear alkenes between C_21_ and C_31_ (Table 1). Both infected and uninfected workers shared the same number of compounds but relative proportions and concentrations changed according to infection and age (Figs 1 and 2, Table S1).

#### a. Quantitative differences of hydrocarbon composition

We found significant differences in the overall hydrocarbon abundance according to infection status and age of workers (LMM: F_3,63_ = 13.31, p < 0.001; Fig. 1). A significant reduction was detected in young infected workers compared to uninfected ones, but no difference was found in the case of old individuals (Fig. 1A, Table S1). Generally, the total abundance was significantly lower for old individuals compared to young ones (Fig. 1A, Table S1).

When considering the three hydrocarbon classes separately, the infection status did not significantly modify the abundance of linear alkanes (LMM: F_1,63_ = 0.69, p = 0.408), only differences between the two age classes were revealed (Fig. 1B, Table S1). For methyl-branched alkanes, a significant decrease was detected for individuals coming from infected colonies (LMM infection: F_1,63_ = 5.36, p = 0.024; Fig. 1C), paired with the already observed decrease in old individuals (LMM age: F_1,63_ = 32.84, p < 0.001). Pairwise comparisons resulted in significant differences only in young workers (Fig. 1C, Table S1). Abundance of the alkenes was similar to that of the alkanes. It was also not influenced by fungal infection (LMM: F_1,63_ = 1.11, p = 0.297; Fig. 1D), but it was significantly influenced by age (LMM: F_1,63_ = 28.04, p < 0.001). Pairwise comparisons did not result in any significant differences either in young or in old workers (Fig. 1D, Table S1).

The relative proportion of linear alkanes (Fig. 2) significantly increased both in the chemical profile of young and old infected workers compared to their uninfected counterparts (Table S1). However, we detected a decrease of methyl-branched alkanes in the chemical profile of workers with age and infection (LMM: F_3,65_ = 10.66, p < 0.001) due to significant differences only in old individuals. Differences between age classes irrespective of infection status were also significant. For alkenes, no difference was detected either according to infection status (LMM: F_1,65_ = 0.29, p = 0.596) or age classes (LMM: F_1,65_ = 0.08, p = 0.775).

#### b. Principal component analyses of the hydrocarbon composition

**Figure 3:**
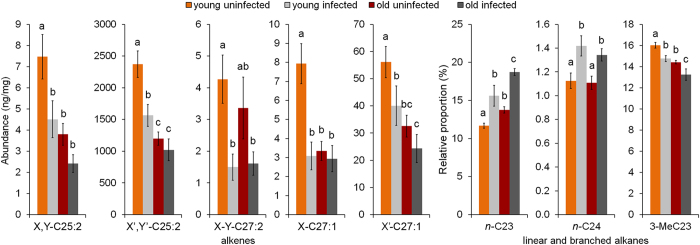
Mean relative proportions (±SE) of specific CHCs of young and old *M. scabrinodis* workers from infected and uninfected colonies. These specific CHCs were pointed out by the principal component analyses for which a fungal effect was detected. Bars with different letters are statistically different according to LMM pairwise comparisons.

When we performed the PCA on the abundances of the CHCs, only the analysis on the alkenes yielded a component which was significantly explained by infection and also by age. In this case, five alkenes showed the highest component loadings (Fig. 3; X,Y-C25:2; X’,Y’-C25:2; X,Y-C27:2; X-C27:1; X’-C27:1), and all showed a significant reduction in young infected workers compared to their uninfected counterparts (LMM: t ≤ 3.87, p < 0.041). In the case of linear alkanes and the methyl-branched alkanes, only age proved to be an explanatory factor (Table 2).

On the basis of the PCA performed on the relative proportions of *n*-alkanes, only for PC2 the best LMM model included the factor infection besides age (Table 2), while PC1 was influenced only by the age of the workers. Among the three peaks with high loadings on the PC2 (Table 2) for which infection proved to have a significant effect, two linear alkanes (*n*-C23 and *n*-C24) had significantly higher relative proportions in infected colonies, and this effect was significant for both young and old workers (LMM: *n*-C23 t ≤ −2.86, p < 0.006; *n*-C24 t ≤ −2.25, p < 0.028; Fig. 3). In uninfected and infected colonies, changes in the third compound, *n*-C25, were only connected with the age of workers (LMM: t ≤ −3.09, p ≤ 0.003).

In the case of methyl-branched hydrocarbons, the PCA returned three components that were significantly explained by the factors tested. The best model for PC3 was explained by both infection and age, while PC1 and PC2 were only explained by age (Table 2). Only the 3-MeC23 showed a significant decrease (Fig. 3) in the relative proportions of the infected colonies for both young (LMM: t = −2.28, p = 0.026) and old (LMM: t = −2.07, p = 0.043) workers.

The PCA on alkenes resulted in three PCs significantly explained by age only (Table 2).

**Figure 4:**
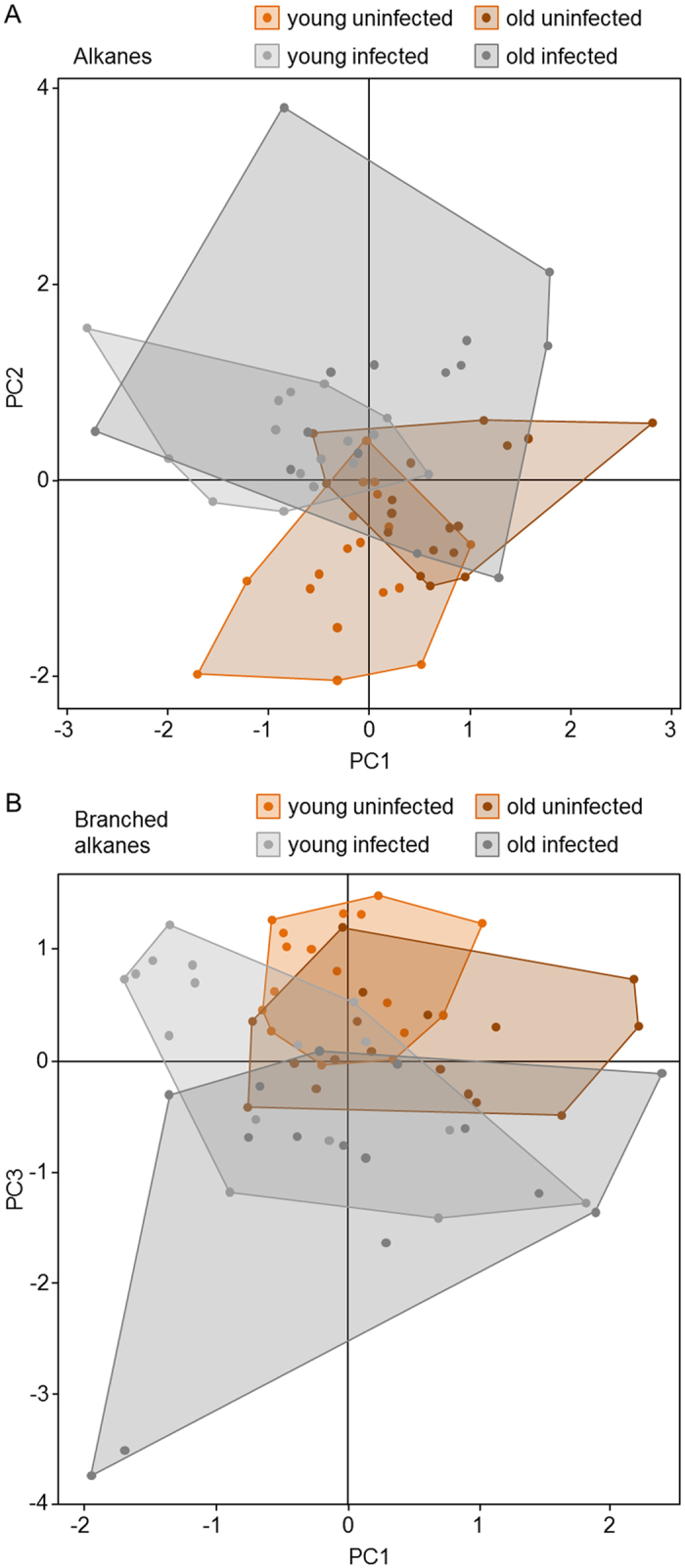
Principal component analysis plots of the CHCs extracted from young and old *M. scabrinodis* workers from uninfected and infected colonies: (**A**) linear alkanes (based on the first and second principal components), and (**B**) methyl-branched alkanes (based on the first and third principal components).

When considering the relative proportion of alkanes, we found variations in the relative dispersion around the centroids for the four worker groups (F = 3.61, p = 0.030; Fig. 4A). A significant increase of variability in the CHC profile was observed for the old workers depending on their infection status (mean Euclidean distance: old UI = 0.817 vs. old I = 1.378).

The methyl-branched alkanes also showed significant differences in the relative dispersion of the samples (F = 4.53, p = 0.010; Fig. 4B). The fungal effect on the variability of methyl-branched alkanes was clear when comparing infected and uninfected workers regardless of their age (mean Euclidean distance: UI = 0.86 vs. I = 1.33) (F = 7.69, p = 0.010; Fig. 4B). These differences were only marginally significant (Fig. S2) when considering separate age classes both for young (mean Euclidean distance: young UI = 0.64 vs. young I = 1.16) and old workers (mean Euclidean distance: old UI = 0.88 vs. old I = 1.37).

This analysis was not performed for alkenes, since infection did not significantly affect either their abundance or their relative proportion.

### Colony structure

The structure of the 18 assessed colonies was highly variable with regards to both worker number (mean no. of workers = 1298.89 ± 1104.03) and queen number (mean = 10.44 ± 16.39). The GLM analysis showed that larger colonies contained more queens (GLM: z = 11.37, p < 0.001), while also infection had a significant, positive effect on the degree of polygyny (GLM: z = 5.013, p < 0.001; Fig. S3).

In the case of the 11 colonies genotyped we found significant heterozygosity excess, after Bonferroni correction, for all loci. With the exception of 3 colonies, the HWE hypothesis was rejected, but significant heterozygosity excess was found in 8 out of 11 colonies. Stuttering and possible null alleles were not detected for any of the loci with the exception of the MP67 locus. The genotypes of this locus were double checked. However, due to almost binomial character of this locus and the general lack of HWE in our data we decided not to correct for possible null allele effect^28^.

Two parameters concerning the genetic structure of the colonies, namely the mean fixation index (*F*_*ST*_) and mean relatedness (Rel), did not differ significantly between infected and uninfected colonies. The F_ST_ was 0.13 for infected and 0.21 for uninfected colonies; (two-sided 10000 permutation test: p = 0.361), whereas Rel was 0.26 for infected and 0.43 for uninfected colonies (two-sided 10000 permutation test: p = 0.317). The pairwise *F*_*ST*_ values between colonies ranged from moderate to high (from 0.05 to 0.4), whereas the mean pairwise within colony relatedness value ranged from 0.1 to 0.7 in the studied colonies.

### The effects of fungal infection on ant behaviour

#### a. Aggression assays between ant workers

**Figure 5:**
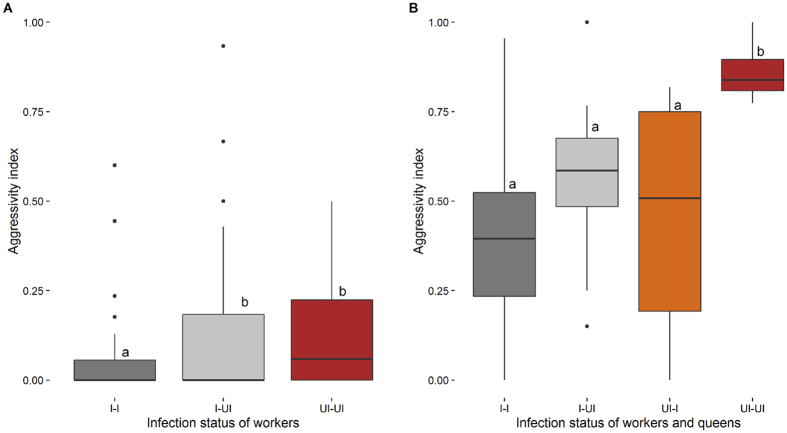
Aggression indices (**A**) between *M. scabrinodis* workers of different infection status, and (**B**) between *M. scabrinodis* workers (w) and queens (q) of different infection status: I – infected by *R. wasmannii*, UI – uninfected (median, quartiles, min-max values).

No aggressive interactions occurred in the control experiments in which aggression between nestmates was tested; only neutral or positive behaviours were recorded. Aggression between non-nestmates was significantly lower when both ant workers were infected (I–I) in comparison to assays in which only one of the workers was infected (I–UI) or both were uninfected (UI–UI) (GLMM: z ≥ 2.47, p ≤ 0.034; Fig. 5A). There was no significant difference between the two latter groups (I–UI vs. UI–UI) (GLMM: z = 0.64, p = 0.519).

#### b. Acceptance of foreign ant queens

The aggression of workers towards foreign queens was significantly higher when both workers and queen came from uninfected colonies (UI–UI) compared to other trials (GLMM: z ≥ 3.27, p < 0.001; Fig. 5B). No differences were found among groups out of which at least one partner (queen and/or workers) was infected by the fungus (I–UI, UI–I, and I–I groups) (GLMM: z ≤ 1.34, p = 0.178).

When the aggression of queens towards workers was analysed, the UI–UI combinations also displayed significantly higher aggression than I–I combinations (GLMM: z = 2.72, p = 0.039), but there were no differences between groups in which at least one partner was infected (I–UI and UI–I groups) (GLMM: z ≤ 1.10, p = 0.267).

#### c. Adoption of Maculinea caterpillars

Altogether 32 caterpillars were adopted during the experiments, while the rest were rejected (9) or undiscovered (22). Infected colonies adopted *Maculinea* larvae at a significantly higher proportion (Fisher’s exact test: p = 0.042). Significant changes (e.g., higher rejection by uninfected) were recorded separately in *M. teleius* (Fisher’s exact test: p = 0.050), whereas only marginally significant differences were found in *M. alcon* (Fisher’s exact test: p = 0.056) (Fig. S4). Regardless of the status of the ant colony, the adoption success of *M. alcon* was significantly higher than that of *M. teleius* (Fisher’s exact test: p < 0.001).

**Figure 6:**
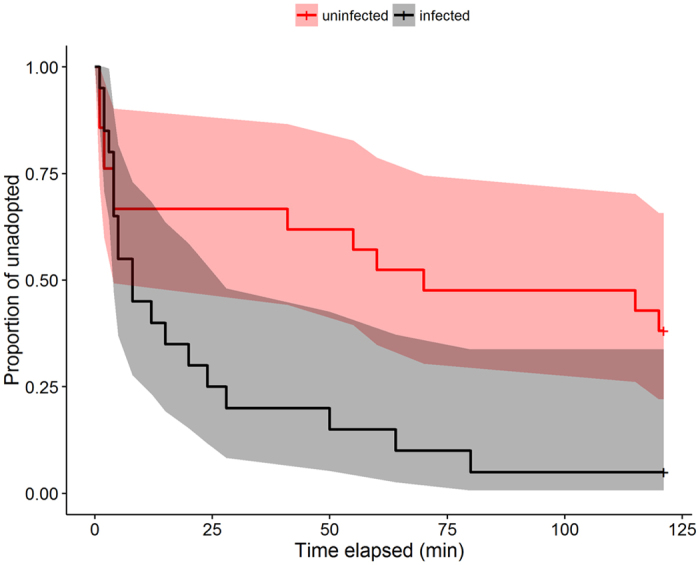
Estimated functions for Cox regression of the adoption time for *Maculinea* caterpillars by ant colonies of different infection status with the point-wise 95% confidence interval for the corresponding functions.

Infected ants adopted caterpillars at a significantly higher rate (Cox coeff = 1.055, χ^2^ = 4.63, p = 0.031; Fig. 6). *Maculinea alcon* larvae were adopted at a significantly higher rate than *M. teleius* (Cox coeff = −1.27, χ^2^ = 17.52, p < 0.001). The interaction of the ant infection status with the species of the caterpillar was not significant (Cox coeff = −0.75, χ^2^ = 0.87, p = 0.350).

There were no significant differences in inspection indices of workers discovering the caterpillars according to the ants’ infection status (GLMM: χ^2^ = 0.1, p = 0.743). The inspection indices did not differ between the two *Maculinea* species (GLMM: χ^2^ = 2.23, p = 0.135), and the interaction between the infection status of the ants and the species of the caterpillar was also not significant (GLMM: χ^2^ = 0.001, p = 0.990).

## Discussion

**Figure 7:**
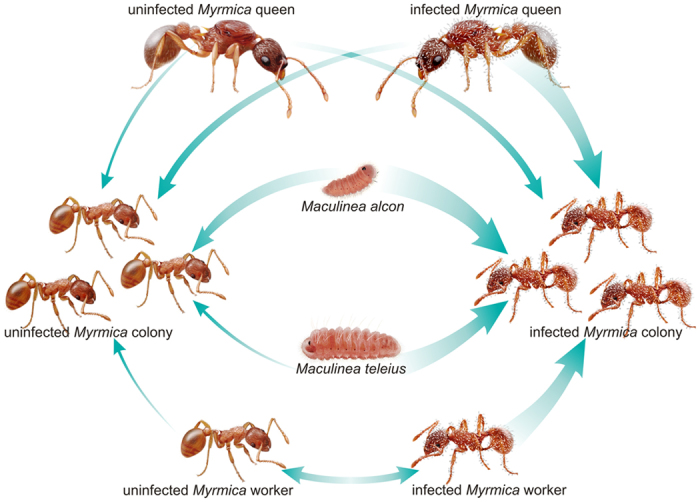
Summary of the experimental setup and the findings of the study. The thickness of arrows correlates with the degree of acceptance by ant colonies of different infection status (drawing by Natalia Timuş).

Our findings indicated that the cues used for nestmate discrimination in *M. scabrinodis* ants were affected by the infection of the ectoparasitic fungus *R. wasmannii*. The CHC profiles of infected workers showed differences in relative proportions of linear and methyl-branched alkanes, and also higher variability, than in uninfected individuals, probably resulting in reduced foe discrimination abilities. Fungus-infected workers were thus less aggressive towards non-nestmates and unrelated queens. As a consequence, chemical and behavioural variations induced by the fungus strengthen the infiltration chances of ‘intruders’, making it easier for strangers (both social parasites and foreign queens) to gain acceptance into the colony (Fig. 7). Therefore, fungal infection, through induced behavioural modifications in individual ants, could affect the fitness of the whole society either positively by (i) increasing genetic heterogeneity, and, implicitly, colony lifespan through higher degree of polygyny, and/or negatively by (ii) decreasing brood quantity through the higher acceptance rate of socially parasitic *Maculinea* larvae. Some of these changes are consistent with the fungus’ need to increase its reproductive and dispersal success, since successful adaptation to hosts with different genetic background, paired with potentially increased colony lifespan, could assist the long-term persistence of the fungus in an invaded host population. In addition, the reduced aggression towards infected queens by uninfected workers could also contribute to the queen’s successful penetration in the new host colonies.

In ants, cuticular compounds play several crucial roles, protecting the animal from water loss and acting as signalling cues primarily as part of the nestmate recognition system^1^,^13^. While uninfected individuals shared a very similar CHC mixture, old infected workers showed much more quantitatively diversified CHC compounds and different proportions of particular linear alkanes and methyl-branched hydrocarbons. According to our findings, these kinds of differences in the CHC profiles cannot be attributed to genetic variation between infected and uninfected colonies, thus the presence or absence of the fungus seems to remain the only plausible explanation.

In general, the presence of fungal parasites can be linked to behavioural, mechanical, biochemical or even to physiological changes in ants^29^,^30^,^31^,^32^. Recently, it was demonstrated that *R. wasmannii* has an influence on the physiology of *M. scabrinodis*, and infected ants in particular spent significantly more time consuming water than uninfected ones^18^. Since the primary function of CHCs is to prevent insect desiccation^1^,^15^, a modification in the abundances and mixture of epicuticular hydrocarbons could increase water loss, thus inducing the higher intake of water observed in infected individuals. Nonetheless, the modification of the CHC composition has the most significant impact on ant recognition abilities. We observed a higher variability in relative proportions of linear and methyl-branched alkanes in old, infected workers, which also correlates with the age-related progress of infection, as old workers are the most infected ones^33^. If we interpret our result in terms of Reeve’s^34^ adjustable threshold model, in *Myrmica scabrinodis* colonies infected by *R. wasmannii* the chemical template gets broader, inducing the infected workers to move towards a more tolerant overall template. Therefore, more acceptance errors will occur in infected colonies than in uninfected colonies. This is consistent with results obtained from our behavioural observations, according to which the aggression level was interpreted as a proxy for acceptance towards non-nestmates. During encounters between non-nestmates which were infected by *R. wasmannii*, the level of aggression decreased compared to encounters in which at least one partner was uninfected, whereas aggression peaked between uninfected non-nestmate workers. This clearly cannot be attributed to any influence of the infection on the general activity of hosts, as recently demonstrated by Csata *et al*.^33^. Bos *et al*.^35^, on the other hand, demonstrated that fungal infection by *Metarhizium brunneum*, which does not modify the CHC profile of hosts, increased the level of aggression towards non-nestmates. One possible explanation for the difference between the outcomes of our study and that of Bos *et al*.^35^ lies in the differences in fungus virulence and host specificity. While *M. brunneum* is a virulent and generalist entomopathogen that kills its hosts quickly^8^, *R. wasmannii* is confined to *Myrmica* ants, and inflicts only small damages to its hosts^18^,^19^,^36^. For *R. wasmannii*, which reproduces by spore transmission among ants, it would be more advantageous to make ants amiable and sociable, thus increasing the frequency of social interactions such as allogrooming, which could efficiently spread the spores. Increased allogrooming indeed occurs more often in *R. wasmannii* infected colonies, as demonstrated by Csata *et al*.^19^.

Making the CHC template used by ants to recognize nestmates broader and less specific also causes a lower level of aggression between infected *Myrmica* workers and unrelated queens. These findings imply that infected colonies could be more open for polygyny, as new queens would be accepted more easily regardless of their infection status. Our data on colony structure indicated that, indeed, infected colonies contain more queens than uninfected colonies. We do not have any data on the effect of *R. wasmannii* on queen fecundity and longevity, but we can presume that, as in the case of workers^19^, fungus infection may decrease life expectancy of queens as well, resulting in higher queen turnover in infected colonies compared to uninfected ones. With ants, the colony foundation success of individual queens is generally around 2% or less^14^. Paradoxically, for a young queen in a population in which some part of the colonies is infected, fungus infection can consequently be beneficial. Even if some life history parameters may decrease due to fungal infection, the reproduction chances due to being successfully adopted by an unrelated colony can be considerably higher when meeting an infected colony. Furthermore, it seems beneficial for the fungus, as it can spread horizontally through workers inside the same colony, but it can also be transmitted vertically by adoption of young infected queens into existing colonies, coming from the same population or even neighbouring populations. According to Hughes *et al*.^36^, we could consider the ant colony rather than the ant individuals as host to *R. wasmannii* where the fungus exploits ant workers and gynes to spread among hosts.

Previous studies also reported modifications in cuticular profiles of organisms in the presence of parasites. In the honey bee/*Varroa* system, a higher production of cuticular hydrocarbons by parasitized compared to unparasitized adult honey bees was observed, while the cuticular profile of parasitized larvae contained higher relative proportions of two unsaturated hydrocarbons, than unparasitized larvae^37^. Modifications in the synthesis/release of 13 CHCs (six *n*-alkanes, five monomethylalkanes, and two dimethylalkanes) were also detected in the CHC profile of the ant *Temnothorax nylanderi* after the infection by the endoparasitic tapeworm *Anomotanoa brevis*, probably explaining the occasional aggressions which the parasitized ants suffer in their own society^38^.

Likewise, in our study we observed a clear effect of *R. wasmannii* infection on two consecutive linear alkanes, *n*-C23 and *n*-C24, which were found in higher relative proportions, and a methyl-branched hydrocarbon, the 3-MeC23, which was negatively affected by the infection. While *n*-C24 represents 1–2% on the overall proportion of cuticular hydrocarbons, *n*-C23 and 3-MeC23 contribute to 25–30% of the chemical profile of workers, suggesting a considerable impact of the fungus on the CHC profile. It is worth noting that these changes are already evident in the chemical profile of young workers, suggesting that the fungus may begin to act even if a few thalli are present on the ant cuticle.

*Rickia wasmannii* infection not only influences intraspecific interactions but it also affects *M. scabrinodis*’ relationships with other organisms, such as socially parasitic *Maculinea* butterfly larvae, which rely on chemical mimicry to get accepted into the host colony^22^,^23^,^26^. If we consider that the chemical profile of newly moulted fourth-instar larvae of all European *Maculinea* species have a simple combination of linear alkanes^22^, we can assume that their adoption may be favoured when encountering *M. scabrinodis* infected foragers (which are all old individuals), less aggressive and characterized by a higher proportion of one or more linear alkanes^39^. Moreover, in a recent study^40^ *n*-C23 and *n*-C24 were shown to represent common hydrocarbons to all cuticular extracts of both IV instar parasitic larvae of *Maculinea nausithous* and its host ant, *Myrmica rubra*, with *n*-C24 being much more abundant in ant brood extracts and able to promote the first contact of *Maculinea nausithous* with *Myrmica rubra* foragers^40^.

According to previous studies^22^,^23^ the quick retrieval of *Maculinea* larvae is supposedly mediated only by the chemical mimicry of surface hydrocarbons on the epicuticle of *Myrmica* workers. Cuckoo larvae, as *Maculinea alcon*, are commonly retrieved in just a few minutes due to higher degree of chemical similarity to their host ants^23^. On the other hand, the retrieval of predatory larvae, such as *M. teleius*, can take much longer^41^. It has been hypothesised that *M. teleius* larvae must exhibit more complex behaviours of adoption, including production of vibroacoustic signals^27^ in order to compensate for the lack of a sophisticated chemical mimicry. Nevertheless, infection of the *Myrmica* host ants by *Rickia wasmannii* seems to assist the adoption of *Maculinea* larvae regardless of their species. Moreover, this effect seems to be quite strong for *M. teleius*, for which the rejection rate of the larvae was higher in uninfected colonies whose discrimination abilities were not compromised by the fungus.

It is important to point out that even though the presence of *R. wasmannii* affected the CHC compositions, we also detected changes in the chemistry of ant cuticles which were age-dependent. Old workers (foragers) have higher proportions of *n*-alkanes and lower proportions of branched alkanes than young ones (mostly nurses), similarly to what is known in other ant species, such as *Pogonomyrmex barbatus*^42^ and *Formica exsecta*^43^.

The scanty knowledge available on the feeding strategy of *R. wasmannii* allows only speculations about the mechanism used by the fungus to modify the ant surface chemistry. However, it is fair to assume that solely by attaching to the cuticle it could change the hydrocarbon profile of its host either by downgrading or by adding components^32^,^44^,^45^. On the other hand, the fungus could also hinder non-nestmate recognition by physically blocking the access of the antennae to the surface of the cuticle or, in advanced cases, even by residing on the antenna. Whatever the mechanism, physical or chemical, in infected colonies the intensity of infection is highly variable. Some individuals, mostly old ones, are covered entirely with fungi, while others, mostly young ones, have only a few or no thalli^12^. Therefore, in infected colonies considerable variation can be expected in sensory detection, and for this reason the acceptance thresholds in infected and uninfected colonies may differ.

Our findings indicated that fungal infection can modify intraspecific behaviour of ants and their interactions with other organisms. Thus, the presence of pathogens can change the outcomes of these interactions and influence the fitness of both the host and its guests. Host behavioural modifications are most probably the result of changes in the discrimination abilities of infected ants, although the precise mechanism responsible for this has not yet been investigated. As suggested by our results, the consequences of parasitic relationships in social contexts could be manifold. Whereas usually changes in individual behaviour are considered, as e.g. the extended phenotype syndromes caused by fluke worms^8^ and *Ophiocordyceps* fungi^11^, we suggest that certain parasites might cause alterations even in the social structure of colonial organisms by making it more susceptible to accept non-kins and other social parasites.

## Materials and Methods

### Study species and site

Experimental *Myrmica scabrinodis* ant colonies, both infected (I) and uninfected (UI), *M. scabrinodis* queens, and socially parasitic *Maculinea* caterpillars were collected from the same grassland area (46.92N, 23.73E, 410–460 m a.s.l., Romania). The colony-level prevalence of the ectoparasitic fungus *Rickia wasmannii* was more than 50% in the *Myrmica scabrinodis* population, while the within-colony prevalence reached 100% in certain colonies^12^. This fungus, like other Laboulbeniales, has no mycelium and its thallus develops from a bicellular ascospore attached to the outer layer of the host cuticle^46^. The fungal infection status of the ants was assessed in the field and confirmed in the lab using an Olympus SZ51 stereomicroscope.

### Analyses of cuticular hydrocarbon profiles

Altogether 6 uninfected and 6 infected *M. scabrinodis* colonies were brought to the laboratory for the analysis of the CHC profiles. Since CHCs can change during insect maturation^47^ both young and old workers were selected randomly from their colonies on the basis of their cuticular pigmentation^48^. For each colony, 5 workers per age category were pooled into a clean glass vial and their CHCs were solvent-extracted using 200 μl of hexane (Sigma) for 20 min after having been weighed. Three replicates were performed for each age class per colony. The extracts were then stored at −20 °C until analysis. Then, workers were dried at 60 °C for 5 days and their dry mass was weighed individually to the nearest 0.0001 mg with an ultra-microbalance (Sartorius SC2).

Prior to chemical analyses, 800 ng of *n*-eicosane (*n*-C_20_; Sigma E-9752) were added to each extract as an internal standard. Samples were then evaporated under a nitrogen flow before being suspended in a final volume of 20 μl of heptane (Sigma). Two μl of each sample were analysed in an Agilent 7890B gas chromatograph coupled with an Agilent 7000C mass spectrometer using a Gerstel MPS autosampler. The GC was equipped with a capillary column 30 m × 250 μm × 0.25 μm (Zebron ZB-5HT INFERNO) using helium as carrier gas at a flow rate of 1 ml/min. Initial program temperature was 70 °C and ramped at 30 °C/min to 150 °C. It was then increased to 320 °C at a rate of 5 °C/min and held for 10 min at 320 °C. Splitless injection (2 mins) was performed with the injector maintained at 280 °C. Mass spectra were acquired in full scan mode every 0.3 s with a scan lapse of 0.1 s over a range of 0 to 600 amu. Electron impact was setup at 70 eV. Mass spectra were analysed by compiling previous publications and comparing fragmentation patterns^49^ with the help of injections of standard series of *n*-alkanes (Fluka, 94234) for calibration. The chromatograms were manually integrated to calculate the area of each peak of interest using the proportion of the sum over the area of all peaks^50^. We also calculated the quantity of CHCs (ng/mg of ant) per worker as a sum of the areas of all the peaks divided by the peak area of the internal standard (*n*-C_20_) and multiplied by 800 (quantities in ng of the internal standard per sample); the resulting value was divided by the weight of the 5-ant sample.

### Colony structure

The structure of colonies used for CHC analyses (with the exception of 1 uninfected) was examined by using microsatellite loci in order to determine whether there were genetic differences between infected and uninfected colonies that could be responsible for any differences in the CHC profiles. In total, 220 individuals from 11 colonies (20 individuals from each) were genotyped; 6 infected and 5 uninfected colonies. In each case the whole colony was collected from the field and workers and queens were counted. In addition to the colonies sampled for DNA analysis we also counted the workers and queens in 3 infected and 4 uninfected colonies. We isolated genomic DNA from the thorax and legs of workers using the Chelex 100 method^51^. Workers were assayed at eight microsatellite markers: Myrt4^52^, MP 67, MP 84^53^, Msca 43, Msca 47, Msca 64, Msca 78^54^, and Msca1^55^. Two sets of multiplex reactions were used with the forward primers labelled with WellRed Dyes D2, D3 and D4 (multiplex 1 – D2: Msca 43; D3: Msca 64, Msca 78; D4: Msca 47; multiplex 2 – D2: MP 84; D3: Myrt 4, Msca 1; D4: MP 67). The PCRs were performed in a total volume of 10 μl composed of 10 ng of DNA template, 0.2 μM of each primer, 5 μl of Multiplex PCR Master Mix (Qiagen) and water. For PCR amplification, a thermal cycler (Applied Biosystems) was used with the following PCR profile: initial denaturation at 95 °C for 15 min (hot start), 40 cycles of 30 s at 94 °C, 90 s at 63 °C (first multiplex) and 60 °C (second multiplex), 90 s at 72 °C, followed by a final elongation step at 72 °C for 10 min. PCR products were genotyped on a CEQ 8000 DNA fragment analyser (Beckman Coulter) and genotypes were scored using the fragment analysis software CEQ^TM^.

### Behavioural assays

Experimental *Myrmica scabrinodis* ant colonies, both infected (I, N = 21) and uninfected (UI, N = 11) were kept in a laboratory in plastic boxes (16 × 10 × 5 cm) with a wet foam brick under controlled conditions (20 °C, 12 L:12D cycle) with a daily food mixture of sugar and proteins^56^.

#### a. Aggression assays between workers

Aggressiveness of workers was tested after four days of acclimatization under laboratory conditions. Ants from 21 infected and 11 uninfected colonies were used in this experiment. As a control, workers from four infected (I) and four uninfected (UI) colonies were used to test the aggressiveness at the intracolonial level. Six repetitions were performed for each tested colony, thus altogether 48 tests were carried out. Only old workers were selected for the purpose of the experiment, since they are mostly located in the periphery of the nest, and are the only which forage outside, thus having the chance of coming across potential intruders, and constituting the first line of colony defence. Therefore, by default their non-kin discrimination ability should be more formed. Selection of old workers was carried out on their within-nest location (in the arena), and on the basis of their cuticular pigmentation, which is traditionally used for age class estimation in *Myrmica* workers^48^. All ants coming from one particular colony were marked on the thorax with the same colour of fast-drying acrylic paint (Paint Royal Talens: ArtCreation essentials). To allow the ants to recover from marking, they were kept in Petri dishes for 30 minutes. Aggression assays were carried out with the use of two connected transparent plastic tubes (3 cm long), which were first separated by a small piece of red plastic foil. A single *Myrmica* worker was placed in each tube. Ants were allowed to acclimatize for one minute before the red plastic foil was removed. After each assay, the plastic tubes were rinsed with ethylic alcohol (98°), and with water, then wiped, and left to dry for at least 20 minutes. Assays were carried out with both infected and uninfected *Myrmica* workers with three replications per treatment (new individuals were used for each test). Altogether, 111 aggression tests were performed in a randomized order with 3 combinations: infected vs. infected (I–I, N = 39), infected vs. uninfected (I–UI, N = 57) and uninfected vs. uninfected (UI–UI, N = 15). The observations started from the first contact of the workers and lasted for three minutes. All interactions were recorded and categorized as: (1) allogrooming, (2) antennation, (3) frightened off, (4) mandible gaping, (5) biting, (6) dragging, and (7) stinging. Allogrooming was considered as positive event, antennation was considered neutral, and the last five behaviours were considered aggressive. An aggression index (AI) for each encounter was calculated as: AI = the total number of aggressive behaviours divided by the total number of interactions^57^.

#### b. Queen acceptance assays

The queen acceptance experiments were carried out concurrently with aggression assays. Twenty-four old workers were collected per colony and put into a small plastic box (5 × 5 × 4 cm) with coated rims using paraffin to prevent ants from escaping. A wet foam brick was added to serve as a nest. Workers were kept in boxes for 24 hours prior to the experiments. Functional, old queens were separated from their original colonies five hours before being introduced into new colonies. During this time, they were kept in Petri dishes with a wet sponge. A single queen was introduced into each experimental colony. The queen was placed in a plastic tube connected to the plastic nest box from where she could freely enter the arena. The observations began with the first contact between the queen and the workers and lasted 15 minutes. All behavioural events, displayed separately by workers and queens, were recorded, and an aggression index (AI) as described above was calculated. The following combinations were tested according to the infection status of the workers and queens: acceptance of infected queens by (a) 10 infected (I_workers_ − I_queen_) and (b) 10 uninfected (UI_workers_ − I_queen_) colonies, and acceptance of uninfected queens by (c) 9 infected (I_workers_ − UI_queen_) and (d) 8 uninfected (UI_workers_ − UI_queen_) colonies.

#### c. Maculinea adoption assays

To assess whether there are differences in the adoption rate of *Maculinea* caterpillars between infected and uninfected *Myrmica scabrinodis* colonies, we chose caterpillars of two co-occurring *Maculinea* species *M. alcon* (the ‘*pneumonanthe*’ ecotype) and *M. teleius*; two species with different feeding strategies and for which *Myrmica scabrinodis* is a primary host of the population under study^58^,^59^. *Maculinea alcon* and *M. teleius* larvae of pre-adoption stage were obtained by collecting their host plants, *Gentiana pneumonanthe* and *Sanguisorba officinalis*, respectively. Shoots were collected from the end of June until the middle of August and kept in the laboratory in water for 2–3 weeks until the caterpillars reached their pre-adoption maturity and fell off the host plant. Altogether, 63 caterpillars of *Maculinea* forms were used during the experiments: 31 *M. alcon*, and 32 *M. teleius* larvae. Thirteen infected and 9 uninfected *Myrmica scabrinodis* colonies were collected from the field. No *Maculinea* larvae were present in these colonies. They were then divided into 31 and 32 queen-less sub-colonies, each containing 50 workers in addition to 10–15 ant larvae and pupae. Ants were kept in transparent plastic boxes (16 × 10 × 5 cm) under the abovementioned laboratory conditions for at least a week prior the experiment.

*Maculinea* caterpillars were divided between infected and uninfected ant sub-colonies as follows: 15 vs. 16 for *M. alcon*, and 16 vs. 16 for *M. teleius* larvae, respectively. A single fourth-instar larva of *Maculinea* was presented to each ant sub-colony. The caterpillar was placed into the plastic box at the position opposite to the ant shelter. The time elapsed from the introduction of the caterpillar until its discovery by ants and its transportation to the shelter was recorded on a minute by minute basis for 120 minutes. Caterpillars were considered adopted when they were introduced into the nest by workers, whilst those discovered but not introduced into the nest were regarded as rejected. The behaviour of ants toward the caterpillar was also recorded and categorized as follows: (1) antennation, (2) licking the larval secretions and (3) picking up the caterpillar. Antennation is considered neutral behaviour in ants that is also connected to foe discrimination. Therefore, an inspection index was calculated for each caterpillar separately as: the number of antennation events divided by the sum of all behavioural events recorded between ants and caterpillar.

### Statistics

#### a. Cuticular hydrocarbon profiles

The differences among the four groups (young I, young UI, old I, old UI) with regards to the total proportion of linear alkanes, methyl branched alkanes, and alkenes were tested with a linear mixed model (LMM, maximum likelihood fit) with colony ID as a random factor. The same analysis was carried out separately on the concentrations (ng/mg) of the linear alkanes, methyl-branched alkanes, alkenes, and the overall CHC profile.

Considering that the 3 classes of CHCs that compose the cuticular profile of *M. scabrinodis* workers may convey different information, we reduced the number of variables by performing separate Principal Component Analyses (PCA based on correlations, varimax rotation) on linear alkanes (9 variables), methyl-branched alkanes (13), and alkenes (13). We excluded 2 compounds (*n*-C28 + unknown and *n*-C30 + unknown) for which the CHC class was not clearly identified. For each analysis, we retained the principal components with eigenvalues ≥1. LMMs (maximum likelihood fit) were used to investigate the effect of age and infection, and their interaction, on each retained principal component, including the colony ID as a random factor. Best models were selected based on the lowest Akaike’s information criterion (AIC) values. The same procedure was applied to both transformed relative proportions and overall concentrations using ln(x + 1) formula. From the principal components for which an effect of the infection was discovered, we selected CHC peaks with correlation coefficients ranging between 0.6 and 1 (absolute values). We then tested whether the proportions and concentrations of those CHC peaks were influenced by infection and by age using LMMs (maximum likelihood fit), including the colony ID as a random factor. To compare the within-group data dispersion, non-Euclidean distances between objects and group centroids were handled by reducing the original distances to principal coordinates. To test for significance, we used F-tests based on sequential sums of squares obtained from permutations of the principal component scores (99 permutations). A set of confidence intervals on the differences among the mean distance-to-centroid of the levels of the grouping factor with the specified family-wise probability of coverage were created. The intervals were based on the Studentized range statistic, Tukey’s ‘Honest Significant Difference’ method.

LMMs and PCA were carried out with SPSS 21 (IBM). Differences in dispersion of CHC profiles between I and UI ants were analyzed with R v. 3.2.5 (R Development Core Team) using the *betadisper* and *TukeyHSD. betadisper* functions in the *vegan* package^60^, a multivariate analogous of the Levene’s test for comparing group variances^61^. The graphs were carried out using the *ggplot2* R package^62^.

#### b. Colony structure

The effect of colony size (no. of workers) and fungal infection on the number of queens was analysed using a generalized linear model approach (GLM, Poisson error, maximum likelihood fit) with number of workers as input variable and fungal infection as fixed factor.

The genotyped data were checked for amplification errors and presence of null alleles using Micro-checker Version 2.2.3^63^. The conformance with Hardy-Weinberg expectations (HWE) was calculated in Genepop on the Web (v.4.2)^64^ using an exact probability test (Markov chain parameters: 10000 dememorizations, 100 batches, 1000 iterations per batch) with Bonferroni correction, followed by a heterozygosity excess test with same parameters due to found deviation from HWE. Fixation index (F_ST_) and mean pairwise within colony relatedness (Rel; mean across all loci and colony with correction for sample size) were calculated for groups (infected and uninfected colonies), and tested regarding their differences with a two-sided 1000 permutation test in FSTAT (v.2.9.3)^65^. We calculated mean pairwise within colony relatedness for each colony according to the algorithm of Queller and Goodnight^66^ in Kingroup^67^, with allele frequency calculated from the whole dataset, to confirm the results obtained.

#### c. Behavioural assays

Worker-worker and worker-queen aggression indices were analysed using a generalized linear mixed model approach (GLMM, binomial error, maximum likelihood fit) with the colony ID as a random factor and with different fixed factors: I–I, I–UI and UI–UI for the worker aggression experiment and I_w_–I_q_, I_w_–UI_q_, I_q_–UI_w_, UI_w_–UI_q_ for worker-queen interactions. Inspection indexes resulting from the *Maculinea* adoption experiments were analysed with GLMM (binomial error, maximum likelihood fit) with the ant colony ID as a random factor and with different fixed factors: the infection status of the workers, the caterpillar species and their interactions. Only caterpillars discovered within 120 minutes were considered if at least one ant-caterpillar interaction occurred (N = 41). For the *Maculinea* adoption success the proportion of adopted, rejected and undiscovered caterpillars was compared with Fisher’s exact test between I and UI and colonies, and then separately for each species.

*Maculinea* adoption rates were analysed with a Cox regression approach with mixed effects (Efron approximation, N = 63 caterpillars). The time elapsed until the adoption of the caterpillar was included as a dependent variable, whereas the infection status of the ant colony and the butterfly species and their interaction were included as dummy variables. Initial ant colony ID was included as a random factor to handle dependencies.

All statistics were performed using R (v. 3.1.2, R Development Core Team). For Cox regression analyses the *coxme* function in the *coxme* package^68^ was used, while GLM and GLMMs were performed using *glm, glmer* and *glmer.nb* functions in the *lme4* package^69^. *Relevel* function was used in order to carry out sequential comparisons among factor levels when performing Cox regressions and GLMM analyses. Table-wide sequential Bonferroni-Holm correction revealed the exact significance levels among different factor levels in these cases, and also in the case of pairwise Fisher’s exact tests. The graphs were carried out using the *ggplot2* R package^62^.

### Data availability

Data available from the Dryad Digital Repository: http://dx.doi.org/ 10.5061/dryad.dt226^70^.

## Additional Information

**How to cite this article**: Csata, E. *et al*. Lock-picks: fungal infection facilitates the intrusion of strangers into ant colonies. *Sci. Rep.* **7**, 46323; doi: 10.1038/srep46323 (2017).

**Publisher's note:** Springer Nature remains neutral with regard to jurisdictional claims in published maps and institutional affiliations.

## Supplementary Material

Supplementary Materials

## Figures and Tables

**Table 1:** Cuticular hydrocarbons of *Myrmica scabrinodis* with their relative proportion (±SEM) depending on their infection status (infected/uninfected) or their age classes (young/old).

Peak	Hydrocarbon	Relative proportion (±SEM)	F	Infection effect		Age effect	
				Young uninf vs. inf	Old uninf vs. inf	Uninfected young vs. old	Infected young vs. old
1	*n-*C21	0.65 (±0.41)	1.80	—	—	—	—
2	3-MeC21	0.60 (±0.34)	0.32	—	—	—	—
3	*n-*C22	0.71 (±0.27)	0.64	—	—	—	—
4	3-MeC22	0.58 (±0.22)	0.50	—	—	—	—
5	X-C23:1	1.09 (±0.21)	7.84***	−0.87	1.20	0.75	↓4.79***
6	*n-*C23	12.71 (±1.81)	12.07***	↑ −2.86**	↑ −3.61***	↑ −2.80**	↑ −4.05***
7	7-MeC23	0.14 (±0.10)	0.14	—	—	—	—
8	5-MeC23	0.06 (±0.03)	2.85*	−0.89	−0.67	↑ −2.23*	−1.70
9	3-MeC23	15.21 (±1.21)	10.23***	↓2.28*	↓2.07*	↓3.69***	↓3.25**
10	X-C24:1	0.29 (±0.11)	0.57	—	—	—	—
11	X’-C24:1	0.02 (±0.03)	0.97	—	—	—	—
12	*n-*C24	1.12 (±0.24)	2.70*	↑ −2.49*	↑ −2.25*	0.41	0.71
13	8-MeC24	0.51 (±0.24)	1.64	—	—	—	—
14	X,Y-C25:2	0.11 (±0.05)	1.09	—	—	—	—
15	4-MeC24	16.06 (±4.65)	4.66**	0.51	1.41	0.99	↓3.77***
16	X’,Y’-C25:2	35.17 (±3.95)	0.75	—	—	—	—
17	X-C25:1	2.05 (±0.42)	0.52	—	—	—	—
18	X’-C25:1	0.13 (±0.06)	2.08	—	—	—	—
19	*n-*C25	6.44 (±0.82)	8.16***	−1.69	−1.93	↑ −3.092**	↑ −3.26**
20	5-MeC25	0.62 (±0.32)	18.92***	−1.69	−0.68	↓3.00**	↓6.81***
21	3-MeC25	1.94 (±0.41)	4.76**	0.80	1.04	↓2.36*	↓2.79**
22	5,17 di-MeC25	0.11 (±0.06)	1.64	—	—	—	—
23	*n-*C26	0.12 (±0.05)	0.58	—	—	—	—
24	3,9-diMeC25	0.09 (±0.05)	0.15	—	—	—	—
25	X,Y-C27:2	0.08 (±0.07)	2.61	—	—	—	—
26	X-C27:1	0.10 (±0.04)	1.64	—	—	—	—
27	X’-C27:1	0.85 (±0.20)	1.35	—	—	—	—
28	*n-*C27	0.57 (±0.13)	5.23**	1.58	1.54	↑ −2.53*	↑ −2.43*
29	C28 + unknown	0.03 (±0.01)	3.17*	0.13	−2.62*	−1.61	−0.50
30	X-C29:1	0.43 (±0.26)	3.86*	0.88	1.29	↑ −2.65**	−1.77
31	*n-*C29	0.49 (±0.19)	11.87***	0.99	1.39	↑ −4.55***	↑ −3.62***
32	15-, 13-, 11-MeC29	0.15 (±0.10)	2.02	—	—	—	—
33	5,17-diMeC29	0.14 (±0.11)	2.76*	−0.46	0.16	0.81	↓2.75**
34	C30 + unknown	0.02 (±0.01)	0.78	—	—	—	—
35	X-C31:1	0.40 (±0.24)	4.47**	0.93	1.75	↑ −2.92**	−1.58
36	X’-C31:1	0.05 (±0.03)	2.78*	1.06	1.06	−1.83	1.06
37	*n-*C31	0.15 (±0.09)	8.61***	0.49	0.91	↑ −3.97***	↑−3.07**

*t*-values are represented when a significant difference was observed, with “↓” and “↑” that refer to decrease or increase of the examined CHC between groups. *p < 0.05, **p < 0.01, ***p < 0.001.

**Table 2:** Results of the LMM models on the principal components obtained by PCA analyses of the abundances and the relative proportions of CHCs.

	Components (% variance)	Age	Infection	Compounds with component loadings ≥0.6
**Abundance**				
Linear alkanes	PC1 (77%)	6.51*	3.84^(*)^	*n*-C21, *n*-C22, *n*-C23, *n*-C24, *n*-C25, *n*-C26, *n*-C27, *n*-C29, *n*-C31
Methyl-branched alkanes	PC2 (31%)	30.51***	3.81^(*)^	3-MeC23, 4-MeC24, 5-MeC25, 3-MeC25
Linear alkenes	PC1 (57%)	9.49**	11.09***	X-C23:1, X-C24:1, X,Y-C25:2, X’,Y’-C25:2, X-C25:1, X,Y-C27:2, X-C27:1, X’-C27:1, X-C29:1, X-C31:1, X’-C31:1
**Relative proportion**				
Linear alkanes	PC1 (33%)	21.37***	—	*n*-C26, *n*-C27, *n*-C29, *n*-C31
	PC2 (24%)	14.59***	11.94***	*n*-C23, *n*-C24, *n*-C25
Methyl-branched alkanes	PC1 (30%	9.58**	—	3-MeC21, 3-MeC22, 7-MeC23, 5-MeC23, 8-MeC24
	PC2 (28%)	12.63***	—	5-MeC25, 3,9-diMeC25, 15-, 13-, 11-MeC29, 5,17-diMeC29
	PC3 (12%)	28.76***	8.85**	3-MeC23, 4-MeC24, 3-MeC25
Linear alkenes	PC1 (25%)	8.27**	—	X’-C27:1, X-C29:1, X-C31:1, X’-C31:1
	PC3 (13%)	6.87*	—	X’-C24:1, X’-C25:1
	PC4 (11%)	15.91***	—	X-C23:1, X,Y-C25:2

Only PCs explained by age, infection or both factors (retained by the best LMM models) are shown. F-values of the infections status and the age class are reported, as well as the variance explained by each PC. The CHCs whose component loadings resulted to be higher than 0.6 are also listed. (*)p = 0.055, *p < 0.05, **p < 0.01, ***p < 0.001.

## References

[b1] Barbero F. Cuticular Lipids as a Cross-Talk among Ants, Plants and Butterflies. Int. J. Mol. Sci. 17, 1966 (2016). 10.3390/ijms17121966 PMC5187766 27886144

[b2] Strauss S. Y. & Irwin R. E. Ecological and evolutionary consequences of multispecies plant-animal interactions. Annu. Rev. Ecol. Evol. Syst. 35, 435–466 (2004).

[b3] Chamberlain S. A., Bronstein J. L. & Rudgers J. A. How context dependent are species interactions? Ecol. lett. 17, 881–890 (2014). 24735225 10.1111/ele.12279

[b4] Smith N. G. The advantage of being parasitized. Nature 219, 690–694 (1968). 5667056 10.1038/219690a0

[b5] Thomas F., Fauchier J. & Lafferty K. D. Conflict of interest between a nematode and a trematode in an amphipod host: test of the” sabotage” hypothesis. Behav. Ecol. Sociobiol. 51, 296–301 (2002).

[b6] Konrad M., Grasse A. V., Tragust S. & Cremer S. Anti-pathogen protection versus survival costs mediated by an ectosymbiont in an ant host. Proc. R. Soc. Lond. B 282, 20141976 (2015). 10.1098/rspb.2014.1976 PMC4286035 25473011

[b7] Bagnères A.-G. & Lorenzi M. C. In Insect Hydrocarbons: Biology, Biochemistry and Chemical Ecology (eds Blomquist G. J. & Bagneres A. G.) 282–323 (Cambridge University Press, 2010).

[b8] Schmid-Hempel P. Parasites in Social Insects. (Princeton University Press, 1998).

[b9] Espadaler X. & Santamaria S. Ecto- and endoparasitic fungi on ants from the Holarctic region. Psyche 2012, 168478 (2012).

[b10] Hughes D. P. . Behavioral mechanisms and morphological symptoms of zombie ants dying from fungal infection. BMC Ecol. 11, 13 (2011). 21554670 10.1186/1472-6785-11-13 PMC3118224

[b11] Pontoppidan M.-B., Himaman W., Hywel-Jones N. L., Boomsma J. J. & Hughes D. P. Graveyards on the move: the spatio-temporal distribution of dead *Ophiocordyceps*-infected ants. PloS one 4, e4835 (2009). 19279680 10.1371/journal.pone.0004835 PMC2652714

[b12] Markó B. . Distribution of the myrmecoparasitic fungus *Rickia wasmannii* (Ascomycota: Laboulbeniales) across colonies, individuals, and body parts of *Myrmica scabrinodis*. J. Invertebr. Pathol. 136, 74–80 (2016). 26970261 10.1016/j.jip.2016.03.008

[b13] Lenoir A., D’Ettorre P., Errard C. & Hefetz A. Chemical ecology and social parasitism in ants. Annu. Rev. Entomol. 46, 573–599 (2001). 11112180 10.1146/annurev.ento.46.1.573

[b14] Hölldobler B. & Wilson E. O. The Ants. 746 (Springer Verlag, 1990).

[b15] vander Meer R. K. & Morel L. In Pheromone Communication in Social Insects (eds vander Meer R. K., Breed M. D., Winston M. L. & Espelie K. E.) 79–103 (Westview Press, 1998).

[b16] Soroker V. . Hydrocarbon distribution and colony odour homogenisation in *Pachycondyla apicalis*. Insectes Soc. 50, 212–217 (2003).

[b17] Fürst M. A., Durey M. & Nash D. R. Testing the adjustable threshold model for intruder recognition on *Myrmica* ants in the context of a social parasite. Proc. R. Soc. Lond. B 279, 516–522 (2012). 10.1098/rspb.2011.0581 PMC3234545 21715405

[b18] Báthori F., Csata E. & Tartally A. *Rickia wasmannii* increases the need for water in *Myrmica scabrinodis* (Ascomycota: Laboulbeniales; Hymenoptera: Formicidae). J. Invertebr. Pathol. 126, 78–82 (2015). 25620725 10.1016/j.jip.2015.01.005

[b19] Csata E., Erős K. & Markó B. Effects of the ectoparasitic fungus *Rickia wasmannii* on its ant host *Myrmica scabrinodis*: changes in host mortality and behavior. Insectes Soc. 61, 247–252 (2014).

[b20] Witek M., Barbero F. & Marko B. *Myrmica* ants host highly diverse parasitic communities: from social parasites to microbes. Insectes Soc. 61, 307–323 (2014).

[b21] Radchenko A. & Elmes G. Myrmica Ants (Hymenoptera: Formicidae) of the Old World. 789 (Natura Optima Dux Foundation, 2010).

[b22] Akino T., Knapp J. J., Thomas J. A. & Elmes G. W. Chemical mimicry and host specificity in the butterfly *Maculinea rebeli*, a social parasite of *Myrmica* ant colonies. Proc. R. Soc. Lond. B 266, 1419–1426 (1999).

[b23] Nash D. R., Als T. D., Maile R., Jones G. R. & Boomsma J. J. A mosaic of chemical coevolution in a large blue butterfly. Science 319, 88–90 (2008). 18174441 10.1126/science.1149180

[b24] Schönrogge K., Barbero F., Casacci L. P., Settele J. & Thomas J. A. Acoustic communication within ant societies and its mimicry by mutualistic and socially parasitic myrmecophiles. Anim. Behav. doi: 10.1016/j.anbehav.2016.10.031 (2016).

[b25] Witek M. . Interspecific relationships in co-occurring populations of social parasites and their host ants. Biol. J. Linn. Soc. 109, 699–709 (2013).

[b26] Schönrogge K. . Changes in chemical signature and host specificity from larval retrieval to full social integration in the myrmecophilous butterfly *Maculinea rebeli*. J. Chem. Ecol. 30, 91–107 (2004). 15074659 10.1023/b:joec.0000013184.18176.a9

[b27] Sala M., Casacci L. P., Balletto E., Bonelli S. & Barbero F. Variation in butterfly larval acoustics as a strategy to infiltrate and exploit host ant colony resources. PLoS One 9, e94341 (2014). 24718496 10.1371/journal.pone.0094341 PMC3981827

[b28] Dąbrowski M. . Reliability assessment of null allele detection: inconsistencies between and within different methods. Mol. Ecol. Resour. 14, 361–373 (2014). 24119056 10.1111/1755-0998.12177

[b29] Heinze J. & Walter B. Moribund ants leave their nests to die in social isolation. Curr. Biol. 20, 249–252 (2010). 20116243 10.1016/j.cub.2009.12.031

[b30] Hughes D. . From So Simple a Beginning: The Evolution of Behavioral Manipulation by Fungi. Adv. Genet. 94, 437–469 (2016). 27131331 10.1016/bs.adgen.2016.01.004

[b31] Konrad M. . Social transfer of pathogenic fungus promotes active immunisation in ant colonies. PLoS Biol 10, e1001300 (2012). 22509134 10.1371/journal.pbio.1001300 PMC3317912

[b32] Ortiz-Urquiza A. & Keyhani N. O. Action on the surface: entomopathogenic fungi versus the insect cuticle. Insects 4, 357–374 (2013). 26462424 10.3390/insects4030357 PMC4553469

[b33] Csata E., Bernadou A., Rákosy-Tican E., Heinze J. & Markó B. The effects of fungal infection and physiological condition on the locomotory behaviour of the ant Myrmica scabrinodis. J. Insect Physiol (2017). 10.1016/j.jinsphys.2017.01.004 28082084

[b34] Reeve H. K. The evolution of conspecific acceptance thresholds. Am. Nat. 133, 407–435 (1989).

[b35] Bos N., Lefevre T., Jensen A. & D’ettorre P. Sick ants become unsociable. J. Evol. Biol. 25, 342–351 (2012). 22122288 10.1111/j.1420-9101.2011.02425.x

[b36] Hughes D. P., Pierce N. E. & Boomsma J. J. Social insect symbionts: evolution in homeostatic fortresses. Trends Ecol. Evol. 23, 672–677 (2008). 18951653 10.1016/j.tree.2008.07.011

[b37] Salvy M. . Modifications of the cuticular hydrocarbon profile of *Apis mellifera* worker bees in the presence of the ectoparasitic mite *Varroa jacobsoni* in brood cells. Parasitology 122, 145–159 (2001). 11272645 10.1017/s0031182001007181

[b38] Trabalon M., Plateaux L., Péru L., Bagnères A.-G. & Hartmann N. Modification of morphological characters and cuticular compounds in worker ants *Leptothorax nylanderi* induced by endoparasites *Anomotaenia brevis*. J. Insect Physiol. 46, 169–178 (2000). 12770249 10.1016/s0022-1910(99)00113-4

[b39] Elmes G., Akino T., Thomas J., Clarke R. & Knapp J. Interspecific differences in cuticular hydrocarbon profiles of *Myrmica* ants are sufficiently consistent to explain host specificity by *Maculinea* (large blue) butterflies. Oecologia 130, 525–535 (2002). 10.1007/s00442-001-0857-5 28547253

[b40] Solazzo G., Seidelmann K., Moritz R. F. A. & Settele J. Tetracosane on the cuticle of the parasitic butterfly *Phengaris (Maculinea) nausithous* triggers the first contact in the adoption process by *Myrmica rubra* foragers. Physiol. Entomol. 60, 57–64 (2014).

[b41] Witek M. . Development of parasitic *Maculinea teleius* (Lepidoptera, Lycaenidae) larvae in laboratory nests of four *Myrmica* ant host species. Insectes Soc. 58, 403–411 (2011). 21765539 10.1007/s00040-011-0156-z PMC3123462

[b42] Wagner D. . Task-related differences in the cuticular hydrocarbon composition of harvester ants. Pogonomyrmex barbatus. J. Chem. Ecol. 24, 2021–2037 (1998). 10.1023/a:1010408725464 11545372

[b43] Martin S. J. & Drijfhout F. P. Nestmate and task cues are influenced and encoded differently within ant cuticular hydrocarbon profiles. J. Chem. Ecol. 35, 368–374 (2009). 19263166 10.1007/s10886-009-9612-x

[b44] Lecuona R., Riba G., Cassier P. & Clement J. Alterations of insect epicuticular hydrocarbons during infection with *Beauveria bassiana* or *B. brongniartii*. J. Invertebr. Pathol. 58, 10–18 (1991).

[b45] Napolitano R. & Juárez M. P. Entomopathogenous fungi degrade epicuticular hydrocarbons of *Triatoma infestans*. Arch. Biochem. Biophys. 344, 208–214 (1997). 9244399 10.1006/abbi.1997.0163

[b46] Tragust S., Tartally A., Espadaler X. & Billen J. Histopathology of Laboulbeniales (Ascomycota: Laboulbeniales): ectoparasitic fungi on ants (Hymenoptera: Formicidae). Myrmecol. News. 23, 81–89 (2016).

[b47] Bagnères A.-G., Darrouzet E., Landré X. & Christidès J.-P. Endogenous synchronization of the chemical signature of Reticulitermes (Isoptera: Rhinotermitidae) worker termites. Ann. Soc. Entomol. Fr. (n. s.) 47, 202–208 (2011).

[b48] Moroń D., Witek M. & Woyciechowski M. Division of labour among workers with different life expectancy in the ant *Myrmica scabrinodis*. Anim. Behav. 75, 345–350 (2008).

[b49] Nelson D. & Blomquist G. In Waxes: chemistry, molecular biology and functions (ed Hamilton R. J.) 1–90 (The oily press, 1995).

[b50] Meunier J., Delémont O. & Lucas C. Recognition in ants: social origin matters. Plos One 6, e19347 (2011). 21573235 10.1371/journal.pone.0019347 PMC3087756

[b51] Walsh P. S., Metzger D. A. & Higuchi R. Chelex 100 as a medium for simple extraction of DNA for PCR-based typing from forensic material. Biotechniques 10, 506–513 (1991). 1867860

[b52] Evans J. Parentage analyses in ant colonies using simple sequence repeat loci. Mol. Ecol. 2, 393–397 (1993). 8162228 10.1111/j.1365-294x.1993.tb00032.x

[b53] Herbers J. & Mouser R. Microsatellite DNA markers reveal details of social structure in forest ants. Mol. Ecol. 7, 299–306 (1998).

[b54] Henrich K. O., Sander A. C. & Wolters V. & Dauber, J. Isolation and characterization of microsatellite loci in the ant *Myrmica scabrinodis*. Mol. Ecol. Notes 3, 304–306 (2003).

[b55] Zeisset I., Damm Als T., Settele J. & Boomsma J. J. Microsatellite markers for the large blue butterflies *Maculinea nausithous* and *Maculinea alcon* (Lepidoptera: Lycaenidae) and their amplification in other *Maculinea* species. Mol. Ecol. Notes 5, 165–168 (2005).

[b56] Bhatkar A. & Whitcomb W. Artificial diet for rearing various species of ants. The Florid. Entomol. 53, 229–232 (1970).

[b57] Maák I. . Cues or meaningless objects? Differential responses of the ant *Formica cinerea* to corpses of competitors and enslavers. Anim. Behav. 91, 53–59 (2014).

[b58] Czekes Z. . Differences in oviposition strategies between two ecotypes of the endangered myrmecophilous butterfly *Maculinea alcon* (Lepidoptera: Lycaenidae) under unique syntopic conditions. Insect Conserv. Divers. 7, 122–131 (2014).

[b59] Tartally A. & Varga Z. Host ant use of *Maculinea teleius* in the Carpathian-Basin (Lepidoptera: Lycaenidae). Acta Zool. Hung. 54, 257–268 (2008).

[b60] Oksanen J. . Package ‘vegan’. Community ecology package, version 2,9 (2013).

[b61] Anderson M. J. A new method for non‐parametric multivariate analysis of variance. Austral Ecol. 26, 32–46 (2001).

[b62] Wickham H. ggplot2: elegant graphics for data analysis. (Springer Science & Business Media, 2009).

[b63] Van Oosterhout C., Hutchinson W. F., Wills D. P. & Shipley P. MICRO‐CHECKER: software for identifying and correcting genotyping errors in microsatellite data. Mol. Ecol. Notes 4, 535–538 (2004).

[b64] Rousset F. genepop’007: a complete re‐implementation of the genepop software for Windows and Linux. Mol. Ecol. Resour. 8, 103–106 (2008). 21585727 10.1111/j.1471-8286.2007.01931.x

[b65] Goudet J. FSTAT, a program to estimate and test gene diversities and fixation indices (version 2.9.3) http://www.unil.ch/izea/softwares/fstat.html (2001).

[b66] Queller D. C. & Goodnight K. F. Estimating relatedness using genetic markers. Evolution 43, 258–275 (1989). 10.1111/j.1558-5646.1989.tb04226.x 28568555

[b67] Konovalov D. A., Manning C. & Henshaw M. T. KINGROUP: a program for pedigree relationship reconstruction and kin group assignments using genetic markers. Mol. Ecol. Notes 4, 779–782 (2004).

[b68] Therneau T. Coxme: Mixed effects Cox models. *R package version 2,3* (2012).

[b69] Bates D., Maechler M., Bolker B. & Walker S. lme4 package. Linear mixed-effects models using Eigen and S4. *R package version 1* (2014).

[b70] Csata E., Timuş N., Witek M., Casacci L. P., Lucas C., Bagnères A.-G., Sztencel-Jabłonka A., Barbero F., Bonelli S., Rákosy L. & Markó B. Data from: Lock-picks: fungal infection facilitates the intrusion of strangers into ant colonies. DryadDigital Repository. doi: 10.5061/dryad.dt226 (2017). 10.1038/srep46323 PMC5389342 28402336

