# Supplementary material for: Lock-picks: fungal infection facilitates the intrusion of strangers into ant colonies
Source: Sci Rep. 2017 Apr 12;7:46323. doi: 10.1038/srep46323 (PMC5389342; doi:10.1038/srep46323)

Lock-picks: fungal infection facilitates the intrusion of strangers into ant colonies

Authors: Enikő Csata, Natalia Timuș, Magdalena Witek, Luca Pietro Casacci, Christophe Lucas, Anne-Geneviève Bagnères, Anna Sztencel-Jabłonka, Francesca Barbero, Simona Bonelli, László Rákosy, Bálint Markó

Supplementary material

Table S1. Results of the LMM models (*t*-values) regarding the effect of age and infection status on the major CHC classes of *M. scabrinodis* taking into account both their overall abundances and relative proportions. "↓" and "↑" refer to changes of the examined dependent variable between groups (if significant). * $p < 0.05$, ** $p < 0.01$, *** $p < 0.001$

	**Infection effect**		**Age effect**	
	Young uninf vs. inf	Old uninf vs. inf	Uninfected young vs. old	Infected young vs. old
**Abundance**				
Total	↓ 2.44*	1.16	↓ 4.86***	↓ 3.33***
Linear alkanes	1.20	0.56	↓ 4.27***	↓ 2.74***
Methyl alkanes	↓ 3.30**	1.22	↓ 5.39***	↓ 3.08**
Linear alkenes	1.16	0.99	↓ 4.01***	↓ 3.53***
**Relative proportion**				
Linear alkanes	↑ -2.85**	↑ -3.42***	↑ -3.76***	↑ -4.56***
Methyl alkanes	0.96	↓ 2.08*	↓ 2.39*	↓ 4.88***
Linear alkenes	0.93	0.07	0.71	-1.18

Fig. S2. Confidence intervals on the differences between the mean distance-to-centroid of the levels of the grouping factor (age x infection) calculated from the PCA analysis on (**A**) alkanes and (**B**) methyl-brached alkanes. The intervals are based on the Studentized range statistic. Confidence intervals in red, not overlapping with the dashed line, are significant. Confidence intervals in orange, slightly overlapping with the dashed line, are marginally significant.

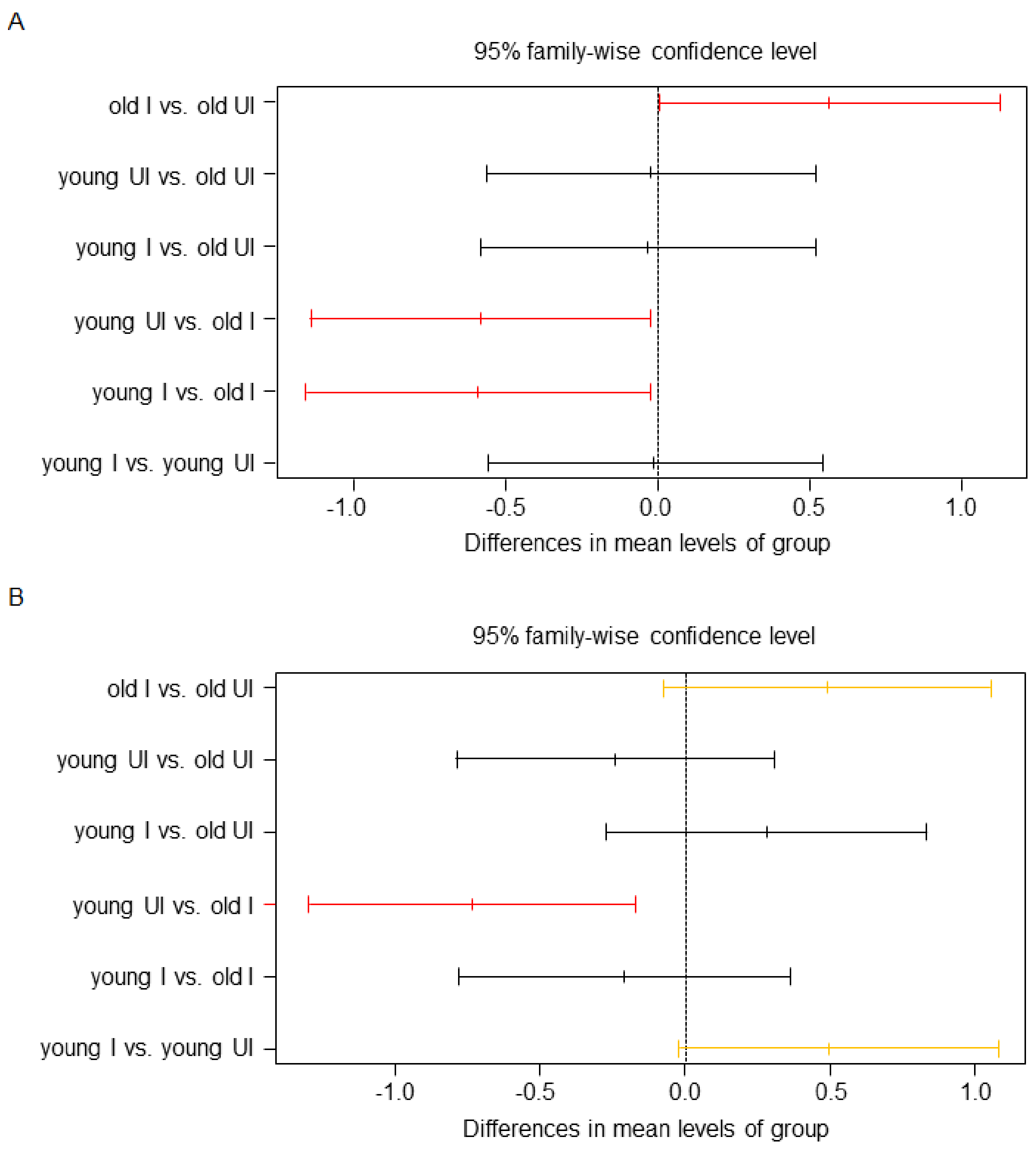

Fig. S3. Number of queens in infected and uninfected colonies (median, quartiles, minimum and maximum values).

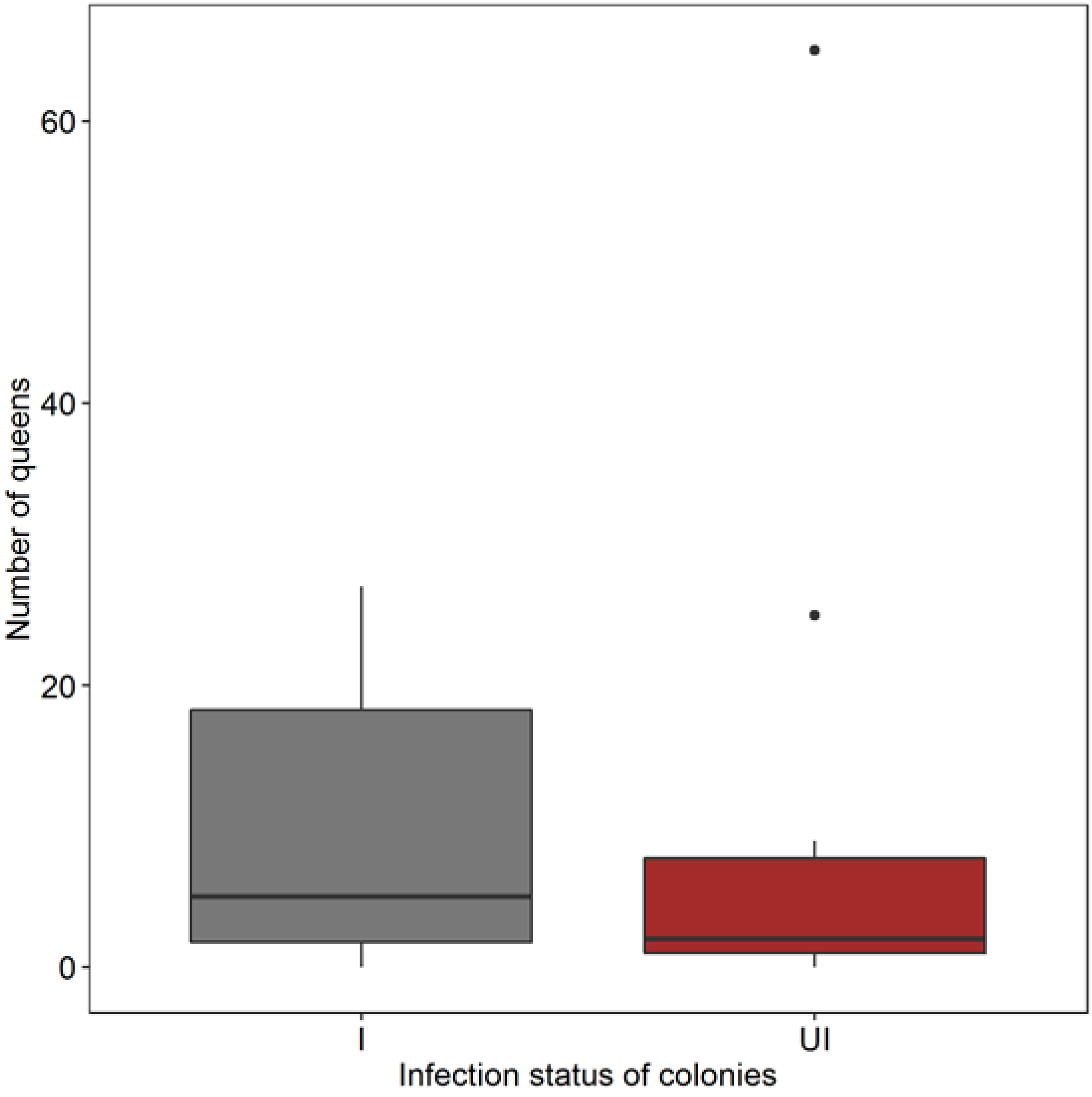

Fig. S4. Number of larvae adopted, rejected and undiscovered per *Maculinea* species, between infected (I) and uninfected (UI) ant colonies.

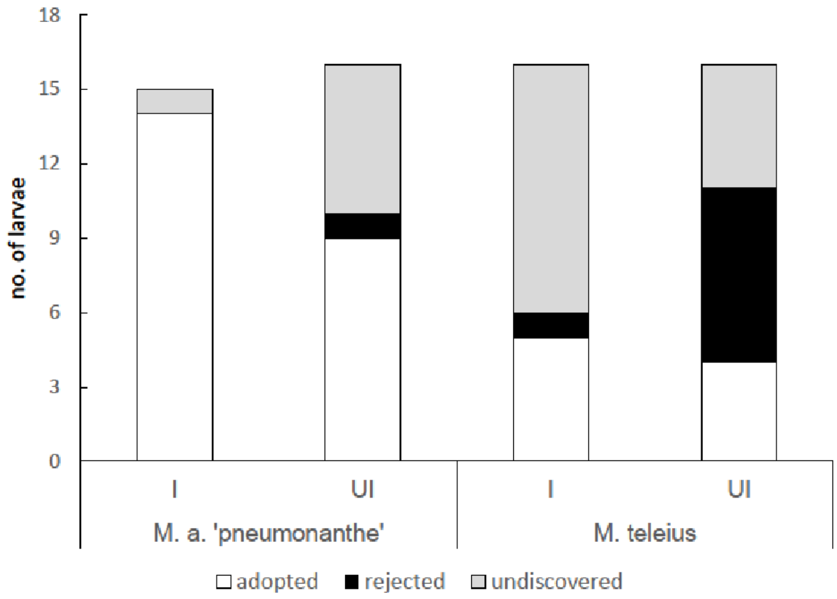

Supplement: Supplementary Materials [file srep46323-s1.pdf]
